# Associations between depressive symptoms, anxiety symptoms, their comorbidity and health-related quality of life: a large-scale cross-sectional study

**DOI:** 10.1186/s12889-021-11969-1

**Published:** 2021-10-21

**Authors:** Wei Liao, Zhicheng Luo, Xiaokang Dong, Xueyan Wu, Yongxia Mei, Ningning Cui, Ning Kang, Yali Lan, Xiaotian Liu, Wenqian Huo, Fang Wang, Chongjian Wang

**Affiliations:** 1 Department of Epidemiology and Biostatistics, College of Public Health, Zhengzhou University, 100 Kexue Avenue, Zhengzhou, 450001 Henan People’s Republic of China; 2 Department of Preventive Medicine, School of Medicine, Henan University of Chinese Medicine, Zhengzhou, Henan P.R. China; 3 Department of Epidemiology, School of Public Health, Shanxi Medical University, Taiyuan, Shanxi P.R. China

**Keywords:** Depressive symptoms, Anxiety symptoms, Comorbidity, HRQoL, EQ-5D-5L, Rural area

## Abstract

**Background:**

There were few studies exploring the associations between depressive symptoms, anxiety symptoms and health-related quality of life (HRQoL) in the general population, especially in resource limited area. The aims of this study were to assess the associations between depressive symptoms, anxiety symptoms, their comorbidity and HRQoL in rural area.

**Methods:**

A total of 23,496 eligible participants from Henan rural cohort were included. The Patient Health Questionnaire-2 (PHQ-2) and Generalized Anxiety Disorder-2 (GAD-2) were employed to assess depressive and anxiety symptoms, respectively. HRQoL was measured via European Quality of Life Five Dimension Five Level Scale (EQ-5D-5L). Tobit regression and generalized linear model were utilized to explore the associations between depressive symptoms, anxiety symptoms, their comorbidity and HRQoL.

**Results:**

A total of 1320 individuals were identified as depressive symptoms with a prevalence rate of 5.62%, while 1198 participants were classified as anxiety symptoms with a prevalence rate of 5.10%. After multiple adjustment, the regression coefficients (*β*) and 95% confidence interval (CI) of utility index for depressive and anxiety symptoms were − 0.166 (− 0.182, − 0.149) and − 0.132 (− 0.149, − 0.114), respectively. The *β* and 95% CI of VAS score for depressive and anxiety symptoms were − 7.65 (− 8.60, − 6.70) and − 5.79 (− 6.78, − 4.80), respectively. Additionally, the comorbidity was strongly associated with low utility index and VAS score. These findings were observed robustly in men and women.

**Conclusion:**

Depressive symptoms, anxiety symptoms and their comorbidity were associated with low HRQoL in rural population, which needed further efforts on preventive and treatment interventions.

**Clinical trial registration:**

The Henan Rural Cohort Study has been registered at Chinese Clinical Trial Register (Registration number: ChiCTR-OOC-15006699). Date of registration: 06 July, 2015.

**Supplementary Information:**

The online version contains supplementary material available at 10.1186/s12889-021-11969-1.

## Background

Mental disorders were major world-wide health problems, which accounted for 13% of the global disease burden, and major depression alone was expected to be the largest contributor by 2030 [1]. In China, the prevalence rates of mental disorders were increasing rapidly, and nearly one in six suffered from mental disorders from 2001 to 2005 [2]. Depression and anxiety were the most prevalent of the many mental disorders, and they often occurred together [3]. Our previous study has shown that the age-standardized prevalence rates of depressive and anxiety symptoms in Chinese rural adults were 5.41% and 4.94%, respectively [4]. In addition, previous studies indicated that depression and anxiety were associated with high prevalence rates of chronic diseases, such as hypertension, type 2 diabetes mellitus (T2DM), and coronary heart disease (CHD) [5–7].

Health related quality of life (HRQoL) was a multidimensional concept contented by physical health status and subjective satisfaction with health, which was a reliable indicator to assess health status [8]. Most previous studies have focused on the effect of physical diseases on HRQoL [9–11], but ignored the effect of mental disorders. Previous studies have explored the associations between depression, anxiety and HRQoL in specific populations [12–14], which indicated that depression and anxiety were associated with low HRQoL. However, there were few studies investigating the associations in the general population, especially in rural population with limited resources. Additionally, multiple studies have documented that women had higher prevalence rates and were approximately twice as likely to suffer from depression and anxiety as men [15, 16]. Thus, it is important to assess gender specific associations between depression, anxiety and HRQoL. Furthermore, depression and anxiety often occurred together [3], but it was still unknown whether suffering from both depression and anxiety will further reduce HRQoL compared to just suffering depression or anxiety only. Therefore, the association between their comorbidity and HRQoL should be examined.

The current study was conducted to explore the associations between depressive symptoms, anxiety symptoms, their comorbidity and HRQoL in rural population. In addition, the difference between men and women was assessed. This study can provide scientific evidence for improving the HRQoL of Chinese rural population.

## Methods

### Study population

The current study, designed as a cross-sectional study, was embedded in Henan rural cohort study, which was a population-based study with a large sample of rural people living in Yuzhou, Suiping, Tongxu, Xinxiang and Yima counties of Henan province in China. From July 2015 to September 2017, 39,259 participants aged from 18 ~ 79 were included in the cohort study via multistage stratified cluster sampling method, with a response rate of 93.7%. Detailed information of the cohort has been described in previous publication [17].

Participants were excluded from the study if they had any of the following: (1) Did not participate in the EQ-5D-5L questionnaire survey (*n* = 15,700); (2) Missing EQ-5D-5L data (*n* = 49); (3) Missing depressive and anxiety symptoms data (*n* = 14). Finally, a total of 23,496 participants were included in this study.

The Henan Rural Cohort Study was approved by the Zhengzhou University Life Science Ethics Committee and conducted in accordance with the principles of the Declaration of Helsinki (Code: [2015] MEC (S128)). Before the study commenced, participants were informed of the study’s purpose, health benefits, and potential hazards. Participants were required to provide informed consent and both the researchers and respondents agreed to use the data for scientific research purposes only.

### Data collection

All participants were interviewed face-to-face by well-trained research staff via a standard questionnaire to collect data including information on demographic characteristics, lifestyle factors and individual history of chronic diseases. To ensure the accuracy and integrity of the collected data, trained investigators checked the integrity and logical errors of the questionnaire on the same day of questionnaire completion, and if there were any problems, they contacted the participants by phone and amended the responses.

Demographic covariates included gender, age, marital status, educational level, and average monthly income. Education level was divided into three levels: elementary school or below, junior high school, and senior high school or above. Average monthly income was also classified into three levels: < 500 RMB, 500–999 RMB and ≥ 1000 RMB. Lifestyle factors covered smoking (never, former, and current), alcohol drinking (never, former, and current) and physical activity. The definition of current smoking and current drinking have been described previous publish [18]. Physical activity was grouped into three levels (low, moderate, and high) according to the validated Chinese version of the International Physical Activity Questionnaire (IPAQ) [19]. Chronic diseases which included hypertension, dyslipidemia, T2DM, CHD and stroke were collected via physical examination, laboratory tests, or self-reports [20]. Body height and weight of the participants were measured twice with shoes and coats off and the readings were recorded to the nearest 0.1 cm and 0.1 kg, respectively. The average readings of the two measurements were taken for statistical analysis in this study. Body mass index (BMI, kg/m^2^) was calculated as weight (kg) divided the square of height (m).

### Definition of depressive and anxiety symptoms

In this study, the Patient Health Questionnaire-2 (PHQ-2) and Generalized Anxiety Disorder-2 (GAD-2) were performed to assess depressive and anxiety symptoms, respectively. The PHQ-2 and GAD-2 are an abbreviated version of the Patient Health Questionnaire-9 (PHQ-9) and the seven-item generalized anxiety disorder scale (GAD-7), met the demands of busy primary care practice and large population-based surveys. Both two scales were reliable and valid screening tools for depressive and anxiety symptoms in Chinese rural population [21, 22].

Both two scales were consisted of two items and each item was consisted of four levels (not at all = 0, several days = 1, more than half the days = 2, and nearly every day = 3), with a total score ranged from 0 to 6. In this study, a cutoff value of 3 was adopted to identify depressive and anxiety symptoms [22, 23]. In other words, participants who reported a score of 3 or above for PHQ-2 or GAD-2 scales were classified as having depressive or anxiety symptoms, respectively. Comorbidity was defined as suffering from depressive and anxiety symptoms simultaneously.

### Assessment of HRQoL

In this study, HRQoL was assessed by European Quality of Life Five Dimension Five Level Scale (EQ-5D-5 L), a standardized measure of HRQoL developed by the EuroQol Group in order to provide a simple, generic measure of health for clinical and economic appraisal [24]. The five dimensions of EQ-5D-5L were mobility (MO), self-care (SC), usual activities (UA), pain/discomfort (PD) and anxiety/depression (AD) and each dimension was consisted of five levels (no problems, slight problems, moderate problems, severe problems, and extreme problems). The EQ-5D-5L utility index was calculated according to the recently available Chinese value set for the EQ-5D-5L instrument [25]. EQ-5D-5L utility index was calculated by the formula [26] as follows:

Utility = 1 − MO × Ln − SC × Ln − UA × Ln − PD × Ln − AD × Ln (n = 1, 2, 3, 4, 5).The EQ-5D-5L also included a visual analogue scale (VAS), a vertical 0 to 100 point rating scale, which reflects the degree of satisfaction with their health status. The best and worst health states carry a score of 100 and 0, respectively.

### Statistical analysis

Statistical description was presented as frequencies and percentages for categorical variables, while means and standard deviations (SD) were calculated for continuous variables. T-test or Kruskal–Wallis test was performed to compare differences between different groups for continuous variables, while Chi squared test was utilized for categorical variables.

Pearson correlation coefficient and Spearmen correlation coefficient were performed to assess the correlation between PHQ-2/GAD-2 and utility index and VAS score. Multivariate Tobit regression model [27] was performed to explore the associations between depressive symptoms, anxiety symptoms and utility index. The Tobit regression model was chosen because the distribution of the EQ-5D utility was skewed and the utility index was censored at 1. Regression methods that ignore the presence of a ceiling effect, or of censoring in the health status measurements can produce biased coefficient estimates. The Tobit regression model is a frequently used tool for modeling censored variables in econometrics research [27]. Different from utility index, VAS score was not a censored data, ranged from 0 to 100. As the VAS score was skewed distribution, a generalized linear model (GLM) was employed to assess the associations between depressive symptoms, anxiety symptoms and VAS score. Model 1 was unadjusted. Model 2 adjusted age, gender, marital status, education level, average monthly income, physical activity, smoking status, drinking status, and BMI. Model 3 further adjusted hypertension, dyslipidemia, T2DM, CHD and stroke based on model 2.

Data were analyzed using SPSS 23.0 software package (SPSS Institute, Chicago) and STATA 15 for Windows. All *P* values were two-tailed with a statistical significance level of 0.05.

## Results

### Characteristics of study participants

The comparison of characteristics between included and excluded samples were shown in Supplementary Table 1. The excluded sample were older and more likely to be females, have lower educational level, be not married/cohabited, have higher average monthly income, have moderate physical activity, have lower average BMI, and were less likely to smoke currently or have chronic illness. Furthermore, the excluded sample had higher prevalence rates of depressive and anxiety symptoms than the included sample (depressive symptoms: 7.27% vs 5.62%; anxiety symptoms: 7.11% vs 5.10%).

Characteristics of study participants according to depressive and anxiety symptoms were presented in Table 1. A total of 1320 individuals were identified as depressive symptoms with a prevalence rate of 5.62%, while 1198 participants were classified as anxiety symptoms with a prevalence rate of 5.10%. The mean age ± SD of participants (41.59% men and 59.41% women) was 55.28 ± 12.63. Compared with non-depressive group and non-anxiety group, depressive group and anxiety group were more prone to be older, women, low education level, and low average monthly income, and less likely to be married/cohabiting, current smoking, current drinking, and obesity (all *P* < 0.05). The mean (SD) utility index and VAS score of the total sample were 0.954 (0.111) and 78.33 (14.80), respectively. Participants with depressive or anxiety symptoms had lower utility index and VAS score than these without depressive or anxiety symptoms (all *P* < 0.001).

**Table 1:** Characteristics of study participants according to depressive symptoms and anxiety symptoms

Variable	Total (***n*** = 23,496)	Depressive symptoms		***P***	Anxiety symptoms		***P***
		Depressive (***n*** = 1320)	Non-depressive (***n*** = 22,176)		Anxiety (***n*** = 1198)	Non-anxiety (n = 22,298)	
Age (year, mean ± SD)	55.28 ± 12.63	56.40 ± 12.33	55.22 ± 12.64	0.001	56.16 ± 11.81	55.24 ± 12.67	0.009
Women n (%)	13,959 (59.41)	886 (67.12)	13,073 (58.95)	< 0.001	842 (70.28)	13,117 (58.83)	< 0.001
Educational level n (%)							
Elementary school or below	10,110 (43.03)	682 (51.67)	9428 (42.51)	< 0.001	635 (53.01)	9475 (42.49)	< 0.001
Junior high school	8979 (38.22)	459 (34.77)	8520 (38.42)		413 (34.47)	8566 (38.42)	
Senior high school or above	4407 (18.75)	179 (13.56)	4228 (19.07)		150 (12.52)	4257 (19.09)	
Married/cohabiting n (%)	21,195 (90.21)	1157 (87.65)	20,038 (90.36)	0.002	1053 (87.90)	20,142 (90.33)	0.006
Average monthly income n (%)							
< 500 RMB	8680 (36.94)	657 (49.77)	8023 (36.18)	< 0.001	551 (45.99)	8129 (36.46)	< 0.001
500- RMB	7452 (31.72)	325 (24.62)	7127 (32.14)		314 (26.21)	7138 (32.01)	
≥ 1000 RMB	7364 (31.34)	338 (25.61)	7026 (31.68)		333 (27.80)	7031 (31.53)	
Physical activity n (%)							
Low	8113 (34.53)	484 (36.67)	7629 (34.40)	0.215	365 (30.47)	7748 (34.75)	0.009
Moderate	7819 (33.28)	432 (32.73)	7387 (33.31)		430 (35.89)	7389 (33.14)	
High	7564 (32.19)	404 (30.60)	7160 (32.29)		403 (33.64)	7161 (32.11)	
Current smoking n (%)	4707 (20.03)	211 (15.98)	4496 (20.27)	< 0.001	174 (14.52)	4533 (20.33)	< 0.001
Current drinking n (%)	4257 (18.12)	173 (13.11)	4084 (18.42)	< 0.001	146 (12.19)	4111 (18.44)	< 0.001
Chronic disease* n (%)	14,578 (62.04)	844 (63.94)	13,734 (61.93)	0.153	740 (61.77)	13,838 (62.06)	0.855
BMI (kg/m2, mean ± SD)	24.99 ± 3.60	24.67 ± 3.80	25.00 ± 3.59	0.002	24.59 ± 3.59	25.01 ± 3.60	< 0.001
Utility index (mean ± SD)	0.954 ± 0.111	0.829 ± 0.224	0.962 ± 0.095	< 0.001	0.835 ± 0.222	0.961 ± 0.097	< 0.001
VAS scores (mean ± SD)	78.33 ± 14.80	66.71 ± 18.27	79.04 ± 14.25	< 0.001	67.43 ± 18.10	78.94 ± 14.34	< 0.001

*Chronic diseases including hypertension, dyslipidemia, T2DM, CHD and stroke

Abbreviation: SD, standard deviation; RMB, Renminbi; BMI, Body mass index

### Self-reported health problems of EQ-5D-5L

**Fig. 1:**
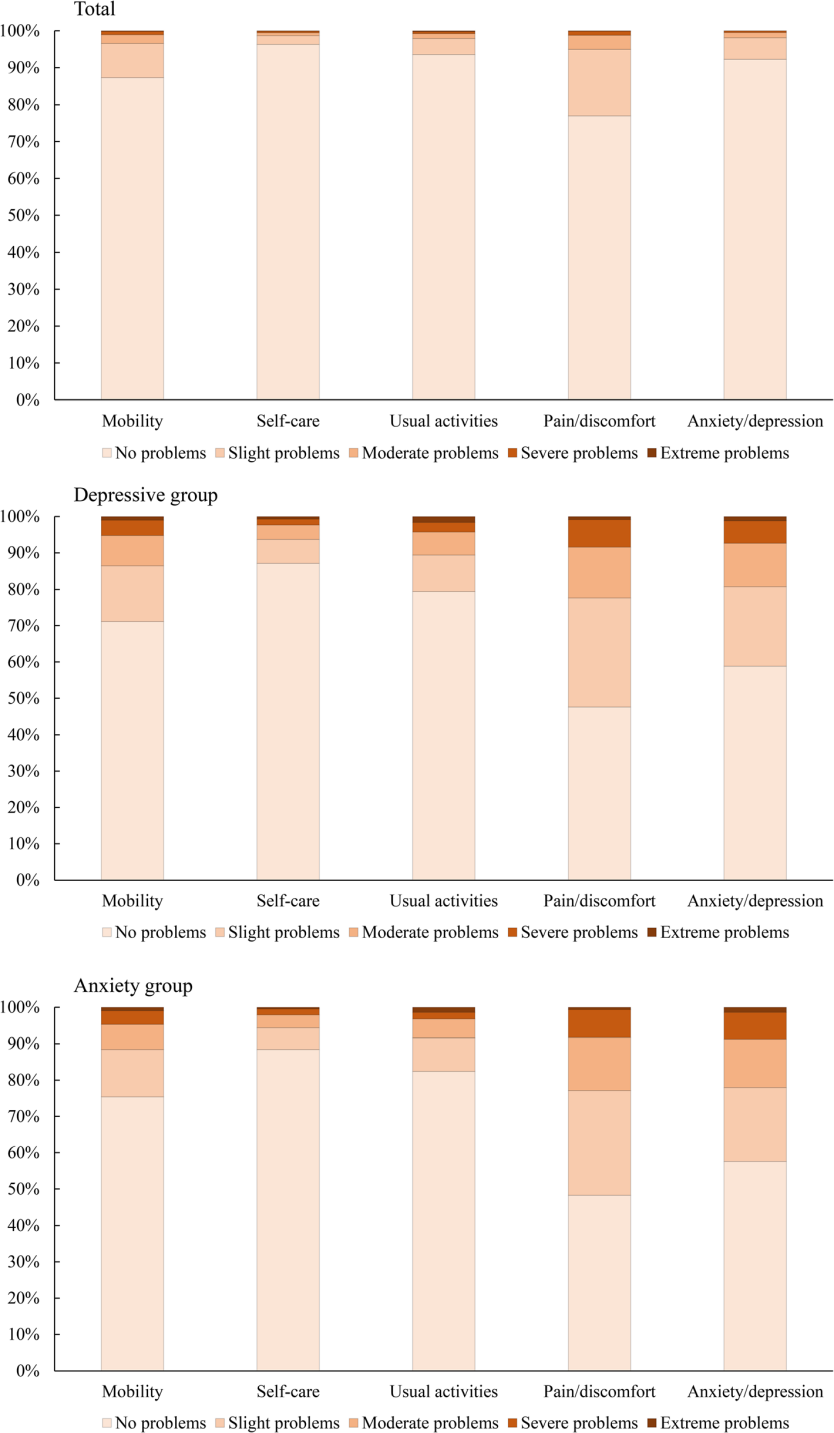
Self-reported health problems of EQ-5D-5L in total sample, depressive group, and anxiety group

Figure 1 demonstrated the self-reported health problems of EQ-5D-5L in total sample, depressive group, and anxiety group. Of all participants, the most frequently reported problem was pain/discomfort dimension (23.03%), followed by mobility (12.72%), anxiety/depression (7.76%), usual activities (6.44%), while the least report was the self-care dimension (3.66%). In depressive group and anxiety group, the most frequently reported problem was still reported in pain/discomfort dimension (52.43% and 51.75%, respectively), followed by anxiety/depression (42.41% and 41.13%, respectively). In both the depressive group and the anxiety group, the percentage of problems reported on all five dimensions increased significantly, especially in anxiety/depression and pain/discomfort dimension. Compared with non-depressive group and non-anxiety group, depressive group and anxiety group had higher percentage of reported health problems in all of five dimensions (all *P* < 0.001). The distribution of utility index and VAS score were shown in Supplementary Figure 1. Both the utility Index and the VAS presented a left-skewed distribution, with the skewness (kurtosis) of − 4.622 (28.998) and − 0.966 (1.585), respectively. The median (interquartile range) of utility index and VAS score were 0.954 (0.058) and 80.00 (20.00), respectively. Although more than half of participants (69.34%) had a utility index of 1, there were still 32 participants reported a utility index less than 0.

### Associations between depressive symptoms, anxiety symptoms and HRQoL

The correlation between PHQ-2/GAD-2 scores and utility index/VAS score were presented in Supplementary Table 2. The Pearson and Spearmen correlation coefficients indicated that PHQ-2/GAD-2 scores were negatively associated with utility index/VAS score (all *P* < 0.001). Supplementary Figure 2 presented the box plots of utility index and VAS score according to PHQ-2/GAD-2 score. The results showed that the utility index and VAS score decreased with the PHQ-2/GAD-2 score increased. The results of Tobit regression and generalized linear models analyses on utility index and VAS score were summarized in Table 2. After multiple adjustments, the Tobit regression model and GLM indicated that both utility index and VAS score were lower in participants with depressive or anxiety symptoms. The regression coefficients and 95% confidence interval (CI) of utility index for depressive and anxiety symptoms were − 0.166 (− 0.182, − 0.149) and − 0.132 (− 0.149, − 0.114), respectively. The regression coefficients and 95% CI of VAS score for depressive and anxiety symptoms were − 7.65 (− 8.60, − 6.70) and − 5.79 (− 6.78, − 4.80), respectively. Compared with participants with PHQ-2 score of 0, the utility index and VAS of participants with PHQ-2 score of 6 decreased 0.201 (− 0.233, − 0.169) and 10.88 (− 12.76, − 8.99), respectively. Similarly, compared with participants with GAD-2 score of 0, the utility index and VAS score of participants with GAD-2 score of 6 decreased 0.191 (− 0.223, − 0.160) and 6.62 (− 8.50, − 4.73), respectively. In addition, the regression analysis also indicated that the utility index and VAS score decreased with the PHQ-2/GAD-2 score increased (all *P*_trend_ < 0.001).

**Table 2:** The results of Tobit regression and Generalized linear models analyses on utility index and VAS score

	Model 1 (***β*** (95% CI))		Model 2 (***β*** (95% CI))		Model 3 (***β*** (95% CI))	
	Utility index	VAS score	Utility index	VAS score	Utility index	VAS score
**PHQ-2 score**						
0	Reference	Reference	Reference	Reference	Reference	Reference
1	−0.051 (− 0.064, − 0.037)	− 2.54 (− 3.21, − 1.86)	− 0.063 (− 0.076, − 0.050)	− 3.43 (− 4.08, − 2.77)	− 0.061 (− 0.073, − 0.048)	− 3.29 (− 3.93, − 2.64)
2	−0.089 (− 0.104, − 0.073)	− 5.66 (− 6.45, − 4.86)	− 0.094 (− 0.108, − 0.080)	−6.20 (− 6.97, − 5.43)	− 0.090 (− 0.104, − 0.076)	−5.94 (− 6.69, − 5.18)
3	−0.177 (− 0.201, − 0.152)	−7.71 (− 9.06, − 6.36)	− 0.165 (− 0.188, − 0.142)	− 7.32 (− 8.63, − 6.01)	− 0.158 (− 0.181, − 0.136)	−6.77 (− 8.05, − 5.48)
4	− 0.168 (− 0.198, − 0.138)	−9.11 (− 10.78, − 7.45)	−0.147 (− 0.176, − 0.119)	−8.72 (− 10.33, − 7.10)	−0.141 (− 0.169, − 0.113)	− 8.35 (− 9.94, −-6.75)
5	−0.248 (− 0.298, − 0.197)	−13.24 (− 16.15, − 10.34)	−0.232 (− 0.279, − 0.185)	− 12.31 (− 15.11, − 9.51)	− 0.220 (− 0.266, − 0.174)	−11.49 (− 14.24, − 8.74)
6	−0.244 (− 0.279, − 0.210)	−13.30 (− 15.28, − 11.32)	− 0.211 (− 0.244, − 0.179)	−11.60 (− 13.51, − 9.69)	− 0.201 (− 0.233, − 0.169)	−10.88 (− 12.76, − 8.99)
*P*_trend_	< 0.001	< 0.001	< 0.001	< 0.001	< 0.001	< 0.001
**Depressive symptoms**						
Yes	−0.194 (− 0.212, − 0.176)	− 9.03 (− 10.01, − 8.04)	− 0.174 (− 0.191, − 0.167)	− 8.22 (− 9.18, − 7.26)	− 0.166 (− 0.182, − 0.149)	−7.65 (− 8.60, − 6.70)
No	Reference	Reference	Reference	Reference	Reference	Reference
**GAD-2 score**						
0	Reference	Reference	Reference	Reference	Reference	Reference
1	−0.048 (− 0.063, − 0.034)	−0.90 (− 1.63, − 0.17)	−0.062 (− 0.076, − 0.049)	−1.69 (− 2.40, − 0.98)	−0.061 (− 0.074, − 0.048)	− 1.55 (− 2.25, − 0.85)
2	−0.095 (− 0.111, − 0.078)	− 3.53 (− 4.39, − 2.66)	− 0.102 (− 0.117, − 0.087)	− 4.10 (− 4.93, − 3.26)	− 0.100 (− 0.115, − 0.085)	− 3.95 (− 4.78, − 3.13)
3	− 0.103 (− 0.130, − 0.076)	−3.99 (− 5.47, − 2.50)	− 0.104 (− 0.130, − 0.080)	−4.19 (− 5.62, − 2.76)	− 0.100 (− 0.125, − 0.075)	−3.88 (− 5.29, − 2.47)
4	−0.116 (− 0.146, − 0.086)	−4.43 (− 6.11, − 2.75)	− 0.122 (− 0.150, − 0.093)	− 4.75 (− 6.38, − 3.13)	−0.124 (− 0.151, − 0.096)	−4.92 (− 6.52, − 3.33)
5	−0.144 (− 0.196, − 0.093)	−4.57 (− 7.52, − 1.64)	− 0.135 (− 0.184, − 0.086)	−4.37 (− 7.21, − 1.52)	− 0.131 (− 0.179, − 0.083)	− 4.03 (− 6.83, − 1.23)
6	− 0.202 (− 0.237, − 0.168)	−6.90 (− 8.88, − 4.92)	− 0.195 (− 0.227, − 0.162)	−7.07 (− 8.99, − 5.16)	−0.191 (− 0.223, − 0.160)	−6.62 (− 8.50, − 4.73)
*P*_trend_	< 0.001	< 0.001	< 0.001	< 0.001	< 0.001	< 0.001
**Anxiety symptoms**						
Yes	−0.139 (− 0.158, − 0.121)	−6.03 (− 7.06, − 4.99)	− 0.135 (− 0.153, − 0.117)	− 6.05 (− 7.05, − 5.05)	− 0.132 (− 0.149, − 0.114)	−5.79 (− 6.78, − 4.80)
No	Reference	Reference	Reference	Reference	Reference	Reference

Abbreviation: CI, confidence interval; PHQ-2, Patient Health Questionnaire-2; GAD-2, Generalized Anxiety Disorder-2

Model 1: Unadjusted

Model 2: Adjusted age, gender, marital status, education level, average monthly income, physical activity, smoking status, drinking status, and BMI

Model 3: Adjusted hypertension, dyslipidemia, T2DM, CHD and stroke based on model 2

**Fig. 2:**
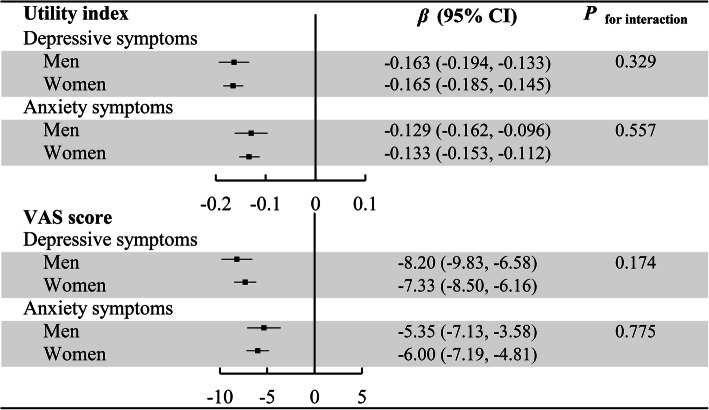
Gender specific associations between depressive symptoms, anxiety symptoms and utility index and VAS score (Adjusted age, gender, marital status, education level, average monthly income, physical activity, smoking status, drinking status, BMI, hypertension, dyslipidemia, T2DM, CHD and stroke)

Figure 2 presented the gender specific associations between depressive symptoms, anxiety symptoms and utility index and VAS score. Both in men and women, the significantly negative associations between depressive, anxiety symptoms and utility index and VAS score was observed. Additionally, there was no interaction between depressive symptoms, anxiety symptoms and gender (all *P*_for interaction_ > 0.05). Supplementary Figure 3 shown the gender specific associations between depressive symptoms only, anxiety symptoms only and utility index and VAS score. Similar results were observed.

### Associations between comorbidity and utility index and VAS score

The comparison of utility index and VAS score between four groups (normal, depressive symptoms, anxiety symptoms and comorbid depressive and anxiety symptoms) were shown in Table 3. The mean (SD) utility index and VAS score of participants with comorbidity were 0.793 (0.243) and 63.70 (18.55), respectively, which were significantly lower than normal group. In addition, participants with depressive symptoms only or anxiety symptoms only were also lower than normal group (all *P* < 0.001). Similar results were observed both in men and women.

**Table 3:** The comparison of utility index and VAS score between four groups (normal, depressive symptoms, anxiety symptoms and comorbid depressive and anxiety symptoms)

	Utility index (mean ± SD)	***P***	VAS (mean ± SD)	***P***
**Total**				
Normal	0.963 ± 0.093	< 0.001	79.13 ± 14.22	< 0.001
Depressive symptoms only	0.877 ± 0.184		70.65 ± 17.22	
Anxiety symptoms only	0.909 ± 0.152		73.80 ± 15.45	
Comorbidity	0.793 ± 0.243		63.70 ± 18.55	
**Men**				
Normal	0.966 ± 0.091	< 0.001	79.17 ± 14.14	< 0.001
Depressive symptoms only	0.893 ± 0.166		69.76 ± 17.38	
Anxiety symptoms only	0.922 ± 0.155		74.59 ± 16.40	
Comorbidity	0.812 ± 0.237		63.81 ± 18.81	
**Women**				
Normal	0.961 ± 0.095	< 0.001	79.10 ± 14.27	< 0.001
Depressive symptoms only	0.869 ± 0.193		71.14 ± 17.13	
Anxiety symptoms only	0.904 ± 0.151		73.49 ± 15.09	
Comorbidity	0.784 ± 0.247		63.66 ± 18.46	

**Fig. 3:**
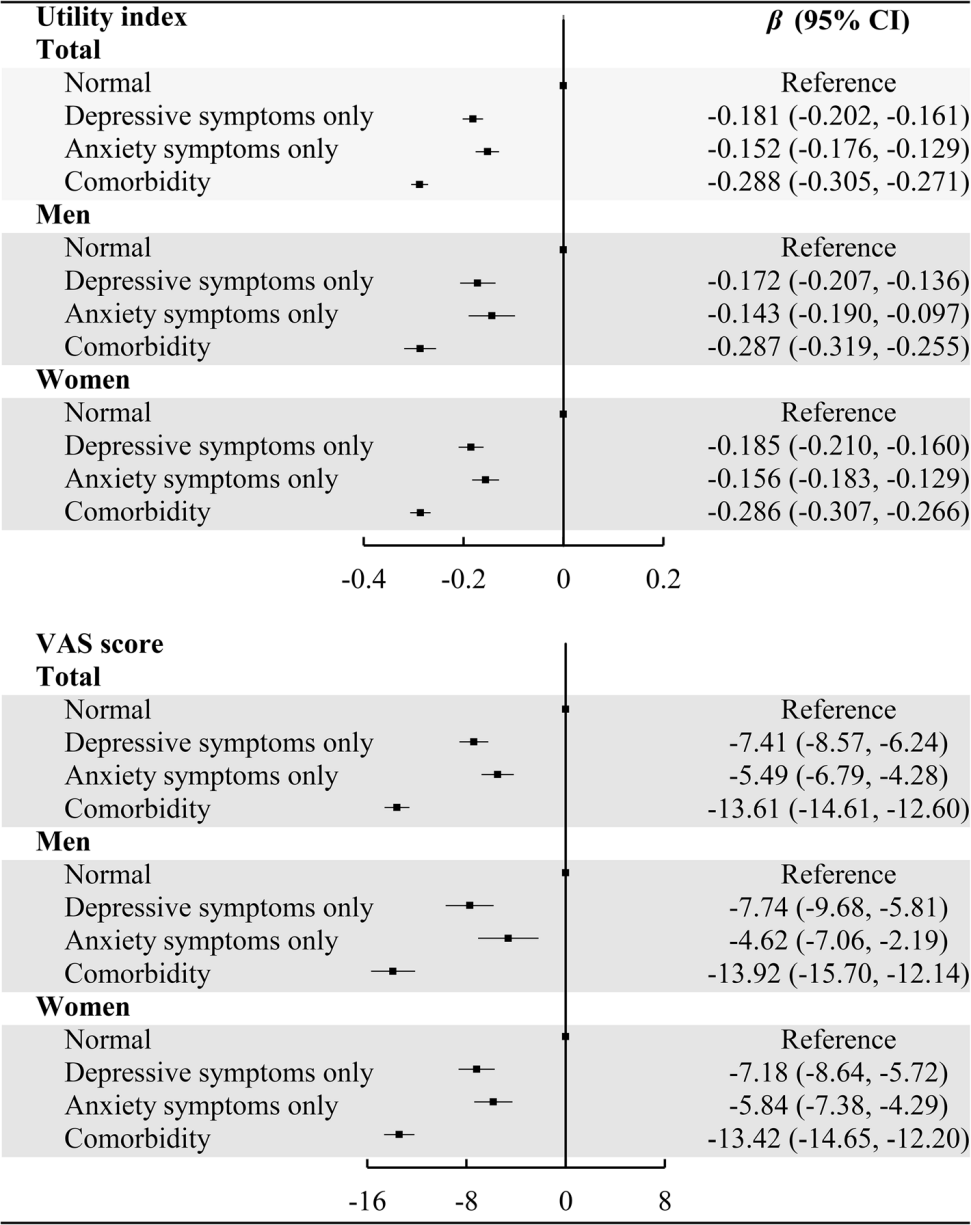
Gender specific associations between comorbidity and utility index and VAS score (Adjusted age, gender, marital status, education level, average monthly income, physical activity, smoking status, drinking status, BMI, hypertension, dyslipidemia, T2DM, CHD and stroke)

The gender specific associations between comorbidity and utility index and VAS score were showed in Fig. 3. The findings indicated that comorbidity was strongly associated with low utility index and VAS score. Of total sample, the regression coefficients and 95% CI for comorbidity in utility index and VAS score were − 0.288 (− 0.305, − 0.271) and − 13.61 (− 14.61, − 12.60), respectively. Notably, the negative associations between depressive symptoms and HRQoL was stronger than anxiety symptoms. These findings were observed robustly in men and women.

## Discussion

In this large population-based study, we investigated the associations between depressive symptoms, anxiety symptoms, their comorbidity and HRQoL, and assessed the difference between men and women. The mean (SD) utility index and VAS score of the total sample were 0.954 (0.111) and 78.33 (14.80), respectively. Of all participants, the most frequently reported problem was pain/discomfort dimension, while the least report was the self-care dimension. The regression analyzes indicated that depressive and anxiety symptoms were associated with low HRQoL. In addition, our results suggested that comorbidity was strongly associated with low HRQoL. Notably, the negative associations between depressive symptoms and HRQoL was stronger than anxiety symptoms. These findings were observed robustly in men and women.

Participants in previous studies conducted in medical institutions had low utility index and VAS score [28, 29]. However, most participants in this study were satisfactory with their HRQoL, which was consistence with the previous studies conducted in China [10, 30]. It may be due to the participants of this study were recruited from rural areas and lived a normal life at home, which hinted HRQoL is better than those who live in medical institutions for professional care. Of all participants, the most frequently reported problem was reported in pain/discomfort dimension, while the least report was the self-care dimension. This was in line with previous studies in China [9, 10]. In both the depressive group and the anxiety group, the percentage of problems reported on all five dimensions increased significantly, especially in anxiety/depression and pain/discomfort dimension. Depressive and anxiety symptoms increased the self-reported problems in pain/discomfort dimension, it may be due to mental disorders and chronic pain tend to further aggravate the severity of both disorders [31]. Self-reported problems in anxiety/depression dimension increased in depressive group and the anxiety group, which indicated that subjectively perceived depression and anxiety were similar to those detected by the PHQ-2 and GAD-2 scale.

In this study, depressive and anxiety symptoms were associated with low HRQoL. Previous studies conducted in specific populations with chronic diseases have found stable associations between mental disorders and low HRQoL [12–14, 32]. In addition, serval studies conducted in old adults also found the negative associations between mental disorders and HRQoL [33–36]. The negative association between depression and HRQoL was also observed in postmenopausal women in Korea [37]. The findings of these studies were consistent with the current study. However, these studies were all conducted in specific subgroup of populations, and may not fully reflect the association between mental health and HRQoL. The current study conducted in general rural population contributed new evidence in rural area and may provide a better understanding of the mental determinants of improving the HRQoL. Nonetheless, it should be noted that our findings based on cross-sectional study cannot confirm causal relationship between depressive symptoms, anxiety symptoms and HRQoL. Certainly, a study suggested that low HRQoL was associated with depression [38].

To the best of our knowledge, this is the first study to investigate the association between comorbidity and HRQoL, whcih suggested that comorbidity was strongly associated with low HRQoL. A prior study conducted in Swedish general population found that comorbidity was associated with higher symptom severity and lower health-related quality of life [39]. However, this study only explored the relationship between comorbidity and each dimension of EQ-5D. The current study has explored the association between comorbidity and utility index calculated according to the recently available Chinese value set, which can better reflect the HRQoL. Our study also found that the negative associations between depressive symptoms and HRQoL was stronger than anxiety symptoms. However, we have not found any other research evidence to support our results and it need further exploration.

The results of this study indicated that depressive and anxiety symptoms were associated with low HRQoL. This may be explained by that individuals with depressive and anxiety symptoms had the following characteristics: fatigue and loss of energy, feeling slowed up or agitated and restless, poor attention and concentration, slow thinking, distractibility, impaired memory, and indecisiveness [40], which will lead to low HRQoL. In addition, depression and anxiety were associated with high prevalence rates of chronic diseases [5–7], which may be another reason why depressive and anxiety symptoms contributed to low HRQoL. Comorbid depression and anxiety can increase impairment and health care use, compared with either disorder alone [40]. Their co-occurrence is often associated with a poor prognosis and significant detrimental impact on functioning in the workplace. This may explain that comorbid depressive and anxiety symptoms was strongly associated with low HRQoL. The current study found that there were no differences between men and women in the associations of depressive symptoms, anxiety symptoms with HRQoL. Although women had higher prevalence rates and were approximately twice as likely to suffer from depression and anxiety as men [15, 16], the influence of depressive and anxiety symptoms on HRQoL was similar in men and women.

There were several limitations in this study. Firstly, the results only can indicate association and cannot establish causal relationship, because this study was cross-sectional design. Prospective studies on mental disorders and HRQoL are needed. Secondly, the PHQ-2 and GAD-2 are useful screening measures rather than diagnostic tools, thus the prevalence rates of depressive and anxiety symptoms may be overestimate, which can induce bias. However, in busy primary care or large population studies, these two scales were quite suitable for saving time while still providing accepted diagnostic performances. Thirdly, some information of participants in this study was collected based on self-reported, but higher test-retest reliability, effective training of study workers and good field implementation will ensure the accuracy and reliability of the information.

## Conclusions

In summary, this study indicated that depressive and anxiety symptoms were associated with low HRQoL. In addition, comorbidity was strongly associated with low HRQoL and the associations between depressive symptoms and HRQoL was stronger than anxiety symptoms. These findings were observed robustly in men and women. The prevalence rates of depressive and anxiety symptoms were high among Chinese rural population [4], which were associated with low HRQoL. Public health institution should formulate interventions for depression and anxiety to improve the HRQoL of Chinese rural population. However, large-scale prospective studies are needed to prove our findings and provide more information about the causal relationship and internal mechanisms of this association.

## Supplementary Information

**Additional file 1 **: **Supplementary Table 1.** Comparison of characteristics between included and excluded samples. **Supplementary Figure 1.** The distribution of utility index and VAS score. **Supplementary Table 2.** The correlation between PHQ-2/GAD-2 scores and utility index/VAS score. **Supplementary Figure 2.** The box plots of utility index and VAS score according to PHQ-2/GAD-2 score. **Supplementary Figure 3.** The Gender specific associations between depressive symptoms only, anxiety symptoms only and utility index and VAS score (Adjusted age, gender, marital status, education level, average monthly income, physical activity, smoking status, drinking status, BMI, hypertension, dyslipidemia, T2DM, CHD and stroke).

## Data Availability

The data analyzed during current study are available from the corresponding author on reasonable request.

## Abbreviations

T2DM: type 2 diabetes mellitus
CHD: coronary heart disease
HRQoL: health related quality of life
IPAQ: International Physical Activity Questionnaire
BMI: body mass index
PHQ-2: Patient Health Questionnaire-2
GAD-2: Generalized Anxiety Disorder-2
PHQ-9: Patient Health Questionnaire-9
GAD-7: seven-item generalized anxiety disorder scale
EQ-5D-5L: European Quality of Life Five Dimension Five Level Scale
MO: mobility
SC: self-care
UA: usual activities
PD: pain/discomfort
AD: anxiety/depression
VAS: visual analogue scale
SD: standard deviations
GLM: generalized linear model
