# Supplementary material for: Associations between depressive symptoms, anxiety symptoms, their comorbidity and health-related quality of life: a large-scale cross-sectional study
Source: BMC Public Health. 2021 Oct 21;21:1911. doi: 10.1186/s12889-021-11969-1 (PMC8532277; doi:10.1186/s12889-021-11969-1)
Supplement: Supplementary file 1 — Additional file 1 : Supplementary Table 1. Comparison of characteristics between included and excluded samples. Supplementary Figure 1. The distribution of utility index and VAS score. Supplementary Table 2. The correlation between PHQ-2/GAD-2 scores and utility index/VAS score. Supplementary Figure 2. The box plots of utility index and VAS score according to PHQ-2/GAD-2 score. Supplementary Figure 3. The Gender specific associations between depressive symptoms only, anxiety symptoms only and utility index and VAS score (Adjusted age, gender, marital status, education level, average monthly income, physical activity, smoking status, drinking status, BMI, hypertension, dyslipidemia, T2DM, CHD and stroke). [file 12889_2021_11969_MOESM1_ESM.docx]

**Supplementary table 1.** Comparison of characteristics between included and excluded samples.

| **Variables** | **Included sample** | **Excluded samples** | *P* |
| --- | --- | --- | --- |
|  | **(n=23496)** | **(n=15763)** |  |
| Age (year, mean ± SD) | 55.28 ± 12.63 | 56.06 ± 11.49 | <0.001 |
| Women n (%) | 13959 (59.41) | 9810 (62.23) | <0.001 |
| Educational level n (%) |  |  |  |
| Elementary school or below | 10110 (43.02) | 7462 (47.34) | <0.001 |
| Junior high school | 8979 (38.22) | 6664 (42.27) |  |
| Senior high school or above | 4407 (18.76) | 1637 (10.39) |  |
| Married/cohabiting n (%) | 21195 (90.21) | 14048 (89.12) | <0.001 |
| Average monthly income n (%) |  |  |  |
| <500 RMB | 8680 (36.94) | 5334 (33.84) | <0.001 |
| 500- RMB | 7452 (31.72) | 5455 (34.61) |  |
| ≥1000 RMB | 7364 (31.34) | 4974 (31.55) |  |
| Physical activity n (%) |  |  |  |
| Low | 8113 (34.53) | 4602 (29.19) | <0.005 |
| Moderate | 7819 (33.28) | 6986 (44.32) |  |
| High | 7564 (32.19) | 4175 (26.49) |  |
| Current smoking n (%) | 4707 (20.03) | 2780 (17.64) | <0.001 |
| Current drinking n (%) | 4257 (18.12) | 2823 (17.91) | 0.427 |
| Depressive symptoms n (%) | 1320 (5.62) | 473 (7.27) | <0.001 |
| Anxiety symptoms n (%) | 1198 (5.10) | 462 (7.11) | <0.001 |
| Chronic disease* n (%) | 14578 (62.04) | 8683 (55.08) | <0.001 |
| BMI (kg/m2, mean ± SD) | 24.99 ± 3.60 | 24.61 ± 3.51 | <0.001 |

*Chronic diseases including hypertension, dyslipidemia, T2DM, CHD and stroke.

Abbreviation: SD, standard deviation; RMB, Renminbi; BMI, Body mass index.


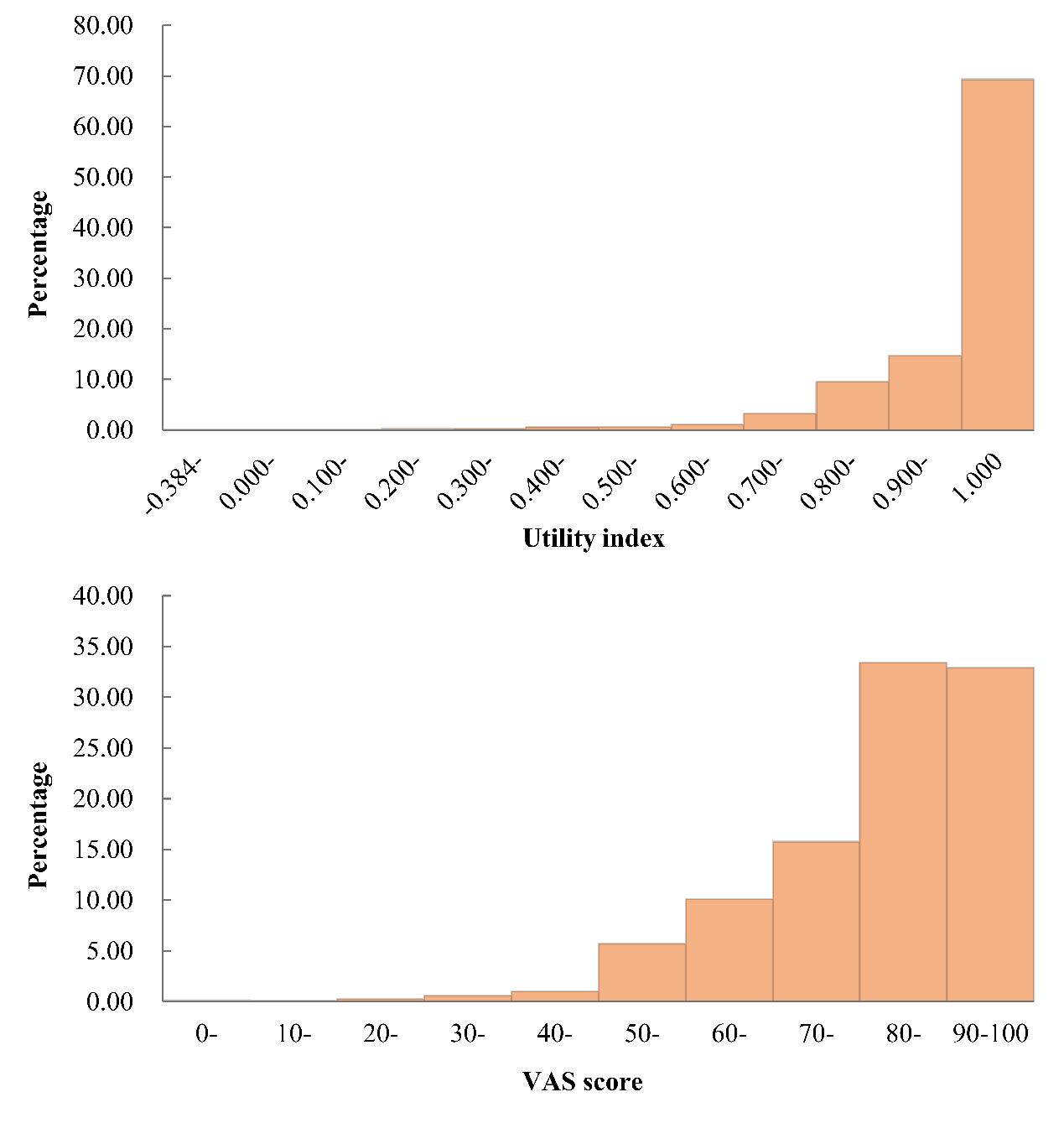


**Supplementary figure 1.** The distribution of utility index and VAS score.

**Supplementary table 2.** The correlation between PHQ-2/GAD-2 scores and utility index/VAS score.

|  | Pearson correlation coefficient | |  | Spearmen correlation coefficient | |
| --- | --- | --- | --- | --- | --- |
|  | Utility index | VAS score |  | Utility index | VAS score |
| PHQ-2 score | -0.307^***^ | -0.247^***^ |  | -0.266^***^ | -0.212^***^ |
| GAD-2 score | -0.296^***^ | -0.219^***^ |  | -0.259^***^ | -0.189^***^ |

^***^*P* <0.001


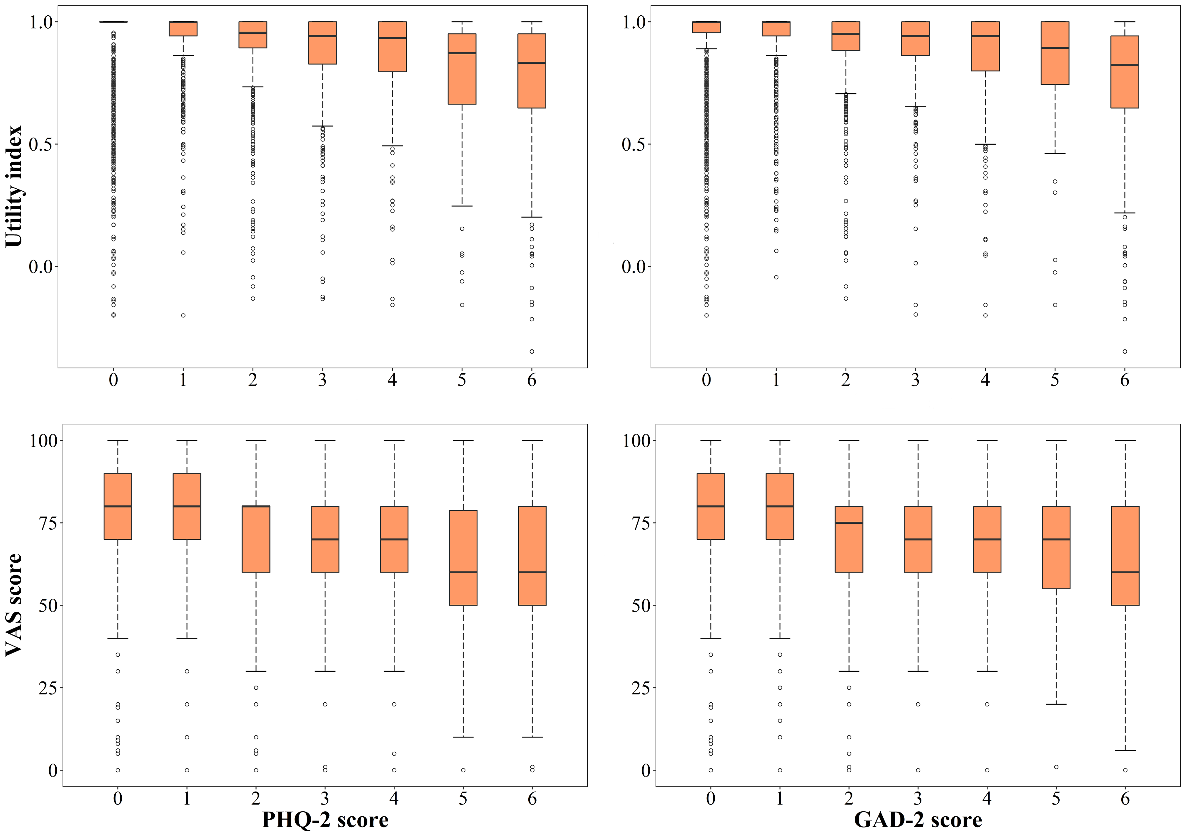


**Supplementary figure 2.** The box plots of utility index and VAS score according to PHQ-2/GAD-2 score.


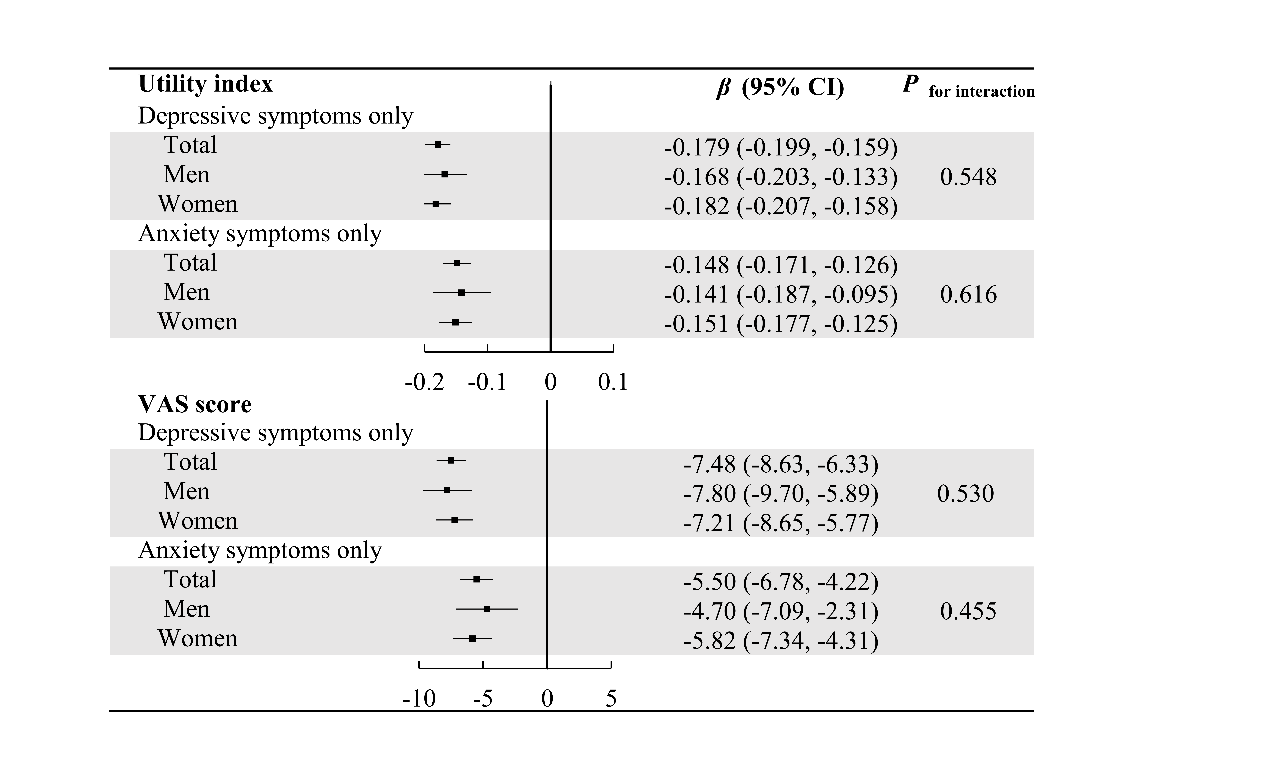


**Supplementary figure 3.** The Gender specific associations between depressive symptoms only, anxiety symptoms only and utility index and VAS score (Adjusted age, gender, marital status, education level, average monthly income, physical activity, smoking status, drinking status, BMI, hypertension, dyslipidemia, T2DM, CHD and stroke).
